# *Dirofilaria immitis* Could Be a Risk Factor for the Development of Allergic Diseases in Humans

**DOI:** 10.3390/ani10101847

**Published:** 2020-10-11

**Authors:** José Alberto Montoya-Alonso, Rodrigo Morchón, Jorge Isidoro Matos, Yaiza Falcón-Cordón, Noelia Costa-Rodriguez, Elena Carretón

**Affiliations:** 1 Research Institute of Biomedical and Health Sciences (IUIBS), University of Las Palmas de Gran Canaria, 35016 Las Palmas de Gran Canaria, Spain; alberto.montoya@ulpgc.es (J.A.M.-A.); jorge.matos@ulpgc.es (J.I.M.); yaiza.falcon@ulpgc.es (Y.F.-C.); noelia.costa101@alu.ulpgc.es (N.C.-R.); 2 Animal and Human Dirofilariosis Group, Laboratory of Parasitology, Faculty of Pharmacy, University of Salamanca, 37007 Salamanca, Spain; rmorgar@usal.es

**Keywords:** *Dirofilaria immitis*, antibodies, Canary Islands, allergic comorbidities, human dirofilariosis, zoonosis

## Abstract

**Simple Summary:**

Heartworm is a parasitic infection transmitted by mosquitoes to animals and humans. The risk of human infections is high in those areas with high canine prevalence, such as the Canary Islands. In these islands, there is also a high number of allergic inhabitants. Since some studies have shown a relationship between parasite infections and development of allergies, the aim of this study was to evaluate whether exposure to heartworm may contribute to the development of allergies. A survey carried out on dog owners in the Canary Islands showed that 51.3% of allergic owners had a heartworm-positive dog, and significant differences were found between allergic or not-allergic owners, according to whether the dog was negative or positive to heartworm. Furthermore, 66 serum samples from inhabitants of the Canary Islands were analyzed for the presence of unspecific allergy markers (Immunoglobulin E or IgE) and specific for heartworm (IgE against *Dirofilaria immitis* and *Wolbachia*) and the results show that people who were in contact with heartworm showed total IgE and specific IgE against heartworm more frequently. Contact with heartworm may be a risk factor for the development of allergic diseases, although further studies to elucidate the relationship between heartworm and allergies should be carried out.

**Abstract:**

The Canary Islands are hyperendemic for canine heartworm and the risk of zoonotic infection is high. Additionally, there is evidence of development of allergies due to nematode infections in animals and humans. Since the population of the Canary Islands presents high prevalence of allergic comorbidities, and previous studies have shown a possible relationship between allergies and seropositivity to heartworm, the aim was to evaluate whether exposure to heartworm may contribute to the development of allergies in the human population. First, an epidemiological study was conducted on 248 owners of dogs with/without heartworm infection in the Canary Islands. Secondly, a serological analysis of the presence of total IgE and specific IgE against heartworm was conducted in 66 samples of inhabitants of the Canary Islands. The survey showed that allergic owners had heartworm-positive dogs more frequently (*p* < 0.01). Of the analyzed human samples, 43.9% were seropositive to heartworm and *Wolbachia*. Total IgE concentrations were increased in 34.5% seropositive samples and 8.1% seronegative samples. Specific IgE against heartworm was only found in human seropositive samples (17.2%). Contact with heartworm may be a risk factor for the development of allergic diseases, although further studies to elucidate the relationship between heartworm and allergies should be carried out.

## 1. Introduction

Heartworm disease, caused by *Dirofilaria immitis*, is a vector-borne disease that affects canids and felids. The domestic dog is the main reservoir of the parasite and the most studied host. Heartworm is a cosmopolitan disease, which is mainly established in areas with temperate and tropical areas throughout the world [1]. Different species of culicid mosquitoes (*Culex* spp., *Aedes* spp. and *Anopheles* spp.) act as vectors for *Dirofilaria*. They feed indistinctly on animals and humans, and since heartworm is a zoonotic disease, inhabitants of endemic areas are at risk of infection. *D. immitis* is the causative agent of pulmonary dirofilariasis in humans, when preadult worms reach the branches of the pulmonary artery, they embolize and cause spherical granulomatous lung lesions. These lesions are benign and generally asymptomatic and, therefore, generally go unnoticed until detected by imaging techniques, in these cases, they are often mistaken for lung tumors [2].

The Canary Islands are hyperendemic for canine heartworm, showing prevalence above 15% in most of the islands [3], and several studies have demonstrated that inhabitants of the islands are at risk of infection, showing 6.4% of the population antibodies against *D. immitis*, and reaching 9%–12.7% in the islands with higher canine prevalence [4,5,6]. This risk is not uniform in all the Canary Islands and depends on the climate conditions and presence of canine heartworm. Those islands with higher canine prevalence present high percentages of humans with anti-*D. immitis* antibodies; thus, they have been in contact with the parasite and are at risk of infection. On the other hand, the inhabitants of islands with low or non-existent canine heartworm, present very low or no seroprevalence [6].

The climate influences the presence of the parasite, and those areas with vegetation, humidity and high temperatures are optimal for the presence of mosquito vectors and development of the parasite. Such is the case for the islands with higher canine and human seroprevalences of *D. immitis*, while the desertic islands present the lower seroprevalence [3,4,5,6].

Asthma, rhinitis, and eczema can be classified together as an allergic comorbidity cluster [7]; furthermore, allergic comorbidity is considered a multifactorial pathology [7,8]. Risk factors for allergies prevalence include a wide set of environmental, infectious (viral agents), genetic and socio-cultural variables. However, other factors may influence the development of allergies, and in many cases the origin of the allergic process is unknown. 

The prevalence of asthmatic adults in the Canary Islands is significantly higher when compared to the national average (17.2% and 5.7%, respectively) [9,10,11,12]. In children, results are similar and prevalence of asthma (18.4%), rhinitis (40.3%) and allergic dermatitis (35.8%) are higher than the national averages [13]. In general, the prevalence of allergic diseases in the Canary Islands is among the highest globally and it is believed that allergic individuals are being underdiagnosed [11,13].

Although sensitization to house dust mites, pollen, animal epithelia or fungus are some of the main factors explaining the high prevalence of allergic diseases, other factors are involved, such as pollution of the air by dusty winds from Africa due to the geographical location of the islands, and increasing pollen presence due to global warming. On the other hand, the trade winds free the archipelago from excessive urban pollution together with the fact that industrialization is much lower than in other Spanish continental regions [10,14]. However, it is recognized that there are other as-yet-unknown factors that influence the high incidence of these pathologies in the Canary Islands [11,13].

Studies have shown the role of some helminth parasites in the development of allergic processes in humans, and have shown associations between allergic sensitization (i.e., to mites) and seropositivity to parasites, such as *Ascaris lumbricoides*, *Toxocara canis, Trichuris trichiura*, hookworm, and *Schistosoma mansoni* [15,16,17,18,19]. Furthermore, there is evidence that some filariae, such as *D. immitis*, may influence the development of hypersensitivity in people [20,21].

Therefore, this study was designed with the aim of evaluating whether exposure to *D. immitis* may contribute to the development of allergic diseases in the population from the Canary Islands.

## 2. Materials and Methods

### 2.1. Epidemiological Study

For this study, two independent methodologies were developed. First, an epidemiological survey was conducted at the Veterinary Teaching Hospital of the University of Las Palmas de Gran Canaria (Canary Islands) in Gran Canaria, a hyperendemic island [3,5]. The study included clients who went with their dogs to perform a heartworm detection test. In this part of the study, 248 dogs and owners participated from February and November 2018. All dogs were tested for the presence of *D. immitis* antigens (Urano Dirofilaria®, Urano Vet SL, Barcelona, España) following manufacturer’s instructions. Furthermore, owners were asked to fill in a questionnaire based on that from Asher et al., [22] (File S1) about diagnosis of allergic comorbidities among the family members living in the same house as the dog, which were confirmed by a medical professional.

### 2.2. Serological Study

Sixty-six human serum samples from inhabitants of the Canary Islands, obtained from a previous study [6], were selected. Of them, 29 were positive to both anti-*D. immitis* IgG and anti-*Wolbachia* surface proteins (WSP) IgG (Group A), and 37 were seronegative (Group B). Sera were randomly selected from the group of positive samples and from the group of negative samples. Levels of total IgE were determined in all samples in the reference laboratory by using chemoluminescence-ELISA techniques (Service of Allergy of the Eurofins Megalab, Canary Islands, Spain). Seropositivity was considered when total IgE were >150 IU/mL.

All samples were further analyzed for the presence of specific anti-*D. immitis* IgE by non-commercial ELISA test, using adult *D. immitis* antigens. Then, 96-well microplates were coated with 0.8 μg of an extract of *D. immitis* adult worms. All serum samples were analyzed at a 1:100 dilution, respectively, and a goat anti-human IgE (H + L) conjugated to horseradish peroxidase (Thermo-Fisher, Barcelona, Spain) was used at 1:100 dilution. Optical densities (ODs) were measured at 492 nm in an Easy Reader (Bio-Rad Laboratories, Hercules, California, USA). The cut-off (OD = 0.5) was established by calculating the mean value + 3 standard deviations (3 SD) of 12 serum samples from clinically healthy blood donors (negative controls) living in a *D. immitis*-free area.

### 2.3. Ethics Statement

All the owners were informed about the present study and consented to participate. The human serum samples were obtained from a previously published study [6]. The present research was approved by the ethical committee of Veterinary Medicine Service of the University of Las Palmas de Gran Canaria (MV-2018/03 and MV-2019/01) and was carried out in accordance with the current European legislation. 

### 2.4. Statistical Analysis

The data were analyzed using the SPSS Base 20.0 software for Windows. The descriptive analysis of the variables considered was carried out studying the proportions in the qualitative variables. The chi-square test was performed to compare proportions. In all the cases, the significance level was established at *p* < 0.05.

## 3. Results

The results from the epidemiological survey show that 29.4% (73/248) of clients who went to the Veterinary Teaching Hospital with their dog had some allergy based on information referred by the owners. Of them, 28.8% (21/73) owned a heartworm-positive dog and 11.4% (20/175) of non-allergic clients owned heartworm-positive dogs (Table 1). Statistically significant differences (*p* < 0.01) between people allergic or not according to whether the dog was negative or positive to heartworm were reported.

The results from the serological analysis of inhabitants of the Canary Islands show that total IgE concentrations were high (>150 IU/mL) in 34.5% (10/29) of seropositive samples to *D. immitis* and WSP (Group A), and 8.1% (3/37) in the seronegative samples (Group B), the differences between dogs being statistically significant (*p* < 0.05). Median IgE value for Group A was 41.20 IU/mL, with a range of 2.10–1242.00 IU/mL (twenty fifth and seventy fifth percentiles 24.40 and 332.80 IU/mL, respectively); median IgE value for Group B was 22.80 IU/mL, with a range of 3.10–83.00 IU/mL (twenty fifth and seventy fifth percentiles 12.60 and 39.00 IU/mL, respectively).

Five samples from the group A were positive for the presence of IgE against *D. immitis* (IgE concentrations were 0.57, 0.53, 0.70, 0.57 and 0.60 IU/mL). All of them had high total IgE concentrations (ranging from 152.20 to 1242 IU/mL) and constituted 17.2% (5/29) of the samples of this group. No high specific IgE concentrations were found in the group B (*p* < 0.05).

## 4. Discussion

For this study, two independent methodologies were carried out, the purpose of which was to analyze possible indicators of a relationship between exposure to *D. immitis* and a greater presence of manifested allergies or IgE in humans.

Although these results are not indicative of causation but, rather, correlation, and the number of allergic and non-allergic owners was similar among owners with heartworm-infected dogs, the epidemiological study showed higher prevalence of allergic owners living with infected dogs. The serological status of these people was not determined, but previous studies have shown that humans living in those areas in which canine heartworm is more prevalent presented higher presence of IgG against *D. immitis* due to the higher risk of being infected [4,6].

Furthermore, the results obtained in the serological study show a relationship between human seropositivity to anti-*D. immitis* and anti-WSP IgG, and presence significantly higher of total IgE. Moreover, presence of anti-*D. immitis* IgE was demonstrated in inhabitants also seropositive to anti-*D. immitis* IgG. The Canary Islands have a very high incidence of allergic diseases. Although allergic comorbidity is a multifactorial pathology, the existence of other unknown factors that influence the high incidence in the Canaries is recognized [10,11].

The measurement of total IgE in serum may be helpful to establish laboratory parameters for the detection of worm diseases. By stimulating type 2 T-helper cells (Th2), IL-4 is released, which induces the synthesis of both monoclonal and polyclonal IgE [23,24]. Both play an important role in the immunological control of multicellular parasites, and a significant increase in the total IgE concentration in serum was observed for several worm/parasite infections [25,26]. Several parasitic molecules have been implicated in modulating the immune and allergic response of helminth-infected hosts [27]. It has been described that parasite infections can develop a strong response and high levels of IgE in allergic sufferers, i.e., asthma or tropical pulmonary eosinophilia [28,29,30,31]. Moreover, it seems that infections by *A. lumbricoides, T. canis, T. trichiura*, hookworm, and *S. mansoni* can cause allergic reactions in humans [15,16,17,18,19]. This has also been described for different types of human filariasis [32] and specifically regarding heartworm, there is some evidence that indicates that this parasite might also have the same capacity [21,33]. When analyzing the results of the present study, it should also be taken into account that recent references claim that that the correlation between helminth infection and allergies should be interpreted inversely [34,35]. Gazzinelli-Guimaraes et al., [36], for instance, have shown that allergic sensitization coincident with filarial infection drives parasite antigen-specific T cell hyperresponsiveness, indicating that allergies could contribute to better immune response fight against helminths.

Previously, a predominant anti-*D. immitis* IgE response was demonstrated in individuals living in an endemic area [37]. Furthermore, previous studies have shown that seropositivity to *D. immitis* and WSP may cause the presence of bronchoconstriction and bronchospasm in the cat, another imperfect host [38,39]. In the Canary Islands there are favorable climatic conditions for the development of mosquito vectors, which allow high prevalence of canine heartworm. Moreover, high seroprevalence of anti-*D. immitis* IgG in humans has been described, as a consequence of constant exposure to infected mosquitoes [3,6]. *D. immitis* and *Wolbachia* have an important role in the stimulation of the immune and inflammatory response during animal and human dirofilariasis [40,41], the human immune reaction to the parasite infection is associated with elevated levels of Th2 cytokines and IgE, among other physiologic responses aimed to terminate the parasite [32]. Therefore, as with other parasites, the results obtained indicate the possibility that constant contact with infected vectors can stimulate the development of specific IgE against *D. immitis*, and that may contribute as another factor to the development of allergies in inhabitants of hyperendemic regions. 

The results of this study should be interpreted with caution, as it presents a series of limitations to be taken into account, such as a limited number of samples, a low prevalence of positive subjects, and, furthermore, the relation of correlation but no causation between allergic diseases and *D. immitis*, in considering that these diseases are multifactorial.

## 5. Conclusions

Despite the results, this study does not demonstrate that contact with *D. immitis* through the mosquito bite, and the seropositivity to specific anti-*D. immitis* IgG and IgE found in inhabitants of the Canary Islands, are responsible for the development of allergic reactions in the human host. Furthermore, given the limitations of the study, results should be interpreted with caution. More studies must be carried out in which the number of samples must be increased, and it must be combined with other techniques that allow determining if there is a causal relationship. However, the results presented here encourage further studies to determine a possible relationship between exposure to *D. immitis* and the development of allergies, in which measurements of allergen-specific IgE levels in the dog owners as well as heartworm parasitic load in the canines would help to elucidate the mechanism of action of this relationship, if any.

## Figures and Tables

**Table 1:** Results obtained from the interviewed owners. Owners are described as allergic or non-allergic, based on the *D. immitis* status of their dogs (infected or non-infected), which visited the Veterinary Teaching Hospital of the University of Las Palmas de Gran Canaria.

Studied Dogs	Allergic Owners	Non-Allergic Owners	Total
Heartworm-infected dogs	21/248 (8.5%)	20/248 (8.1%)	41/248 (16.6%)
Non-infected dogs	52/248 (21.0%)	155/248 (62.5%)	207/248 (83.5%)
Total	73/248 (29.4%)	175/248 (70.6%)	

## Supplementary Materials

The following are available online at https://www.mdpi.com/2076-2615/10/10/1847/s1, File S1: Questionnaire.
