# Supplementary figures and images for: Dirofilaria immitis Could Be a Risk Factor for the Development of Allergic Diseases in Humans
Source: Animals (Basel). 2020 Oct 11;10(10):1847. doi: 10.3390/ani10101847 (PMC7601753; doi:10.3390/ani10101847)

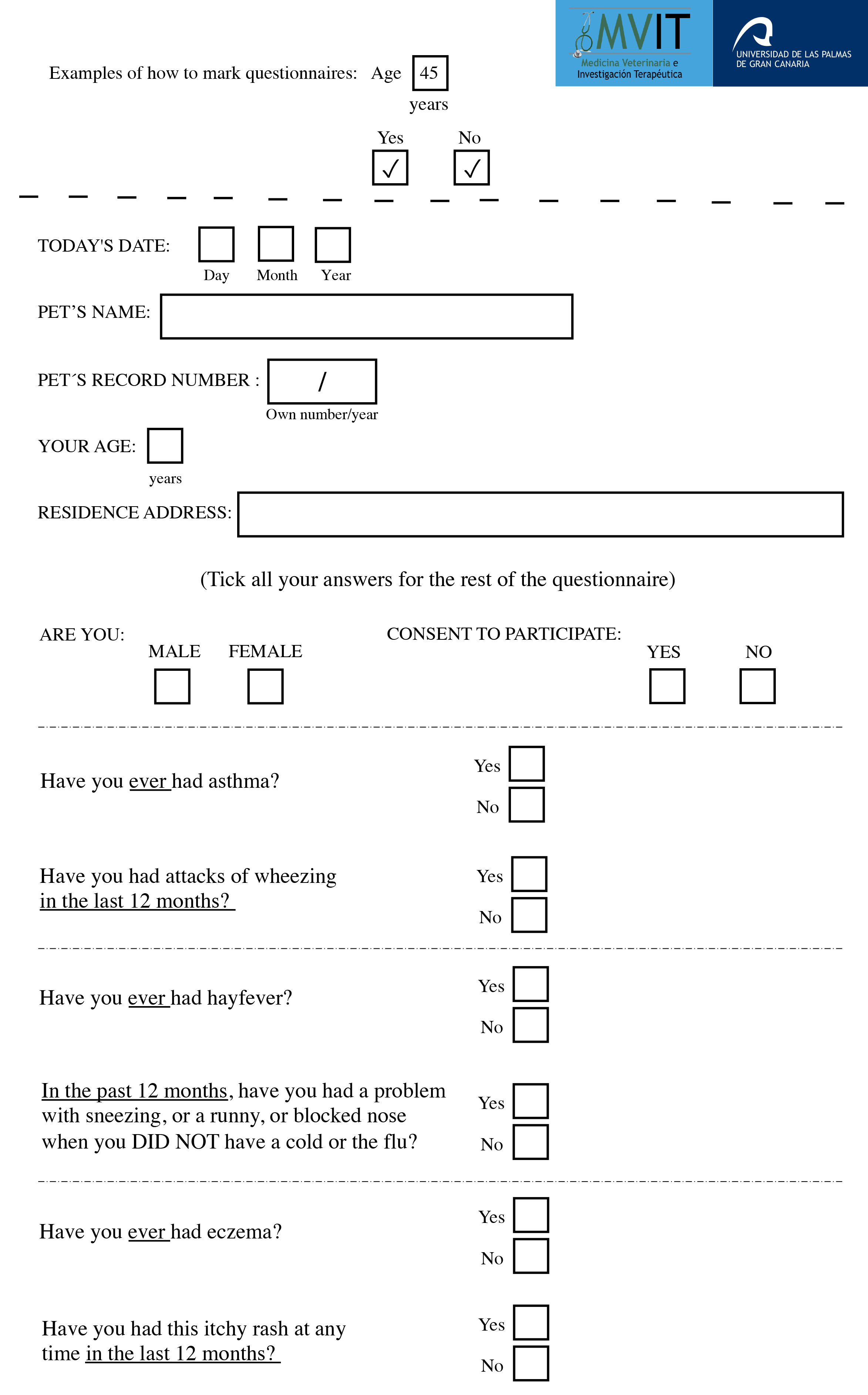

Supplement: Supplementary file 1 [file animals-10-01847-s001.zip › figure 1.tif]
